# Factors associated with the initiation of breastfeeding within the first 48 hours of life in Tabuk, Saudi Arabia

**DOI:** 10.1186/s13006-016-0079-4

**Published:** 2016-07-21

**Authors:** Riyadh A. Alzaheb

**Affiliations:** Department of Clinical Nutrition, Faculty of Applied Medical Sciences, University of Tabuk, Tabuk City, Kingdom of Saudi Arabia

**Keywords:** Breastfeeding initiation, New mothers, Preterm births, Low birth weight, Caesarean section, Public Health Policy

## Abstract

**Background:**

The identification of the factors most closely associated with the initiation of breastfeeding is a vital first step in designing strategies to promote breastfeeding. The study therefore aimed to identify the factors that may be associated with the initiation of breastfeeding in the first 48 h after giving birth among mothers in Tabuk, Saudi Arabia.

**Methods:**

This cross-sectional study was based on a sample of 671 mothers of infants aged up to 24 months at five primary healthcare centers between May and September 2015. A structured questionnaire was used to gather general sociodemographic data along with more detailed information on breastfeeding. A logistic regression analysis was then performed to establish the factors which were independently associated with the mothers’ initiation of breastfeeding.

**Results:**

Breastfeeding was initiated by 92.7 % of mothers within the first 48 h after childbirth. Breastfeeding initiation within the first 48 h of childbirth was lower in women who gave birth by caesarean section (Adjusted Odds Ratio [AdjOR] 0.31, 95 % Confidence Interval [CI] 0.17, 0.57), and had preterm (AdjOR 0.29, 95 % CI 0.12, 0.70) or low birth weight infants (AdjOR 0.35, 95 % CI 0.17, 0.75).

**Conclusions:**

Each variable presents an important barrier to breastfeeding initiation. Suitable hospital policies and staff training are needed to support mothers in quickly initiating breastfeeding, and to discourage the use of infant formula in hospital. To encourage higher rates of exclusive breastfeeding in Saudi Arabia, additional support is required for mothers at a higher risk of failing to initiate breastfeeding in a timely manner.

## Background

The breastfeeding of infants has many benefits, as it provides them with the necessary nutrients for their optimal health and development as well as protecting them against infection [1–3]. Breastfeeding is also important for building a strong biological and emotional foundation for the infant’s health and wellbeing, as well as that of their mother [4–7]. With these benefits in mind, the current recommendation from the World Health Organization (WHO) is that globally, for their first six months of life, infants should be exclusively breastfed [8].

The initiation of breastfeeding is vital to achieving the WHO’s recommendation [9, 10], as it has been found to be associated with longer-term breastfeeding and lower infant mortality, particularly in developing countries [11, 12]. Several factors influence breastfeeding initiation: biomedical, sociodemographic, psychosocial, healthcare, community factors, and government policy. It should be noted that the relative importance of each of these areas differs across different regions and countries and also varies across population sub-groups [13–17]. In order that mothers are able to initiate breastfeeding in the first hours after giving birth, it is important that the factors that influence breastfeeding initiation are identified and understood [18, 19].

In the context of Saudi Arabia, a recent review by Al Juaid et al. (2014) has demonstrated that not enough information has been gathered by prior studies in relation to building an understanding of breastfeeding habits within Saudi Arabia that would enable the measurement of progress, or to plan and design programs to promote and encourage breastfeeding [20]. Unfortunately, no data have been gathered on breastfeeding or, specifically, on the potential factors influencing breastfeeding in Tabuk city (in the North-west region of Saudi Arabia). It is therefore difficult to identify which areas most require intervention among this community.

The aims of the current study are to estimate the occurrence of the initiation of breastfeeding within the first 48 h after childbirth among participating mothers of infants aged up to 24 months at the time of study in Tabuk, Saudi Arabia, and to identify the factors that may have an effect on the initiation of breastfeeding.

## Methods

This cross-sectional study was carried out in Tabuk, Saudi Arabia, between May and September 2015. Tabuk is the largest city in north western Saudi Arabia and the capital of the Tabuk region, and had a population of 534,893 at the 2010 census. The sample size was calculated using the Epi-Info statistical program from the total of 12,760 live births registered in Tabuk in 2014, with a 50 % prevalence assumed of breastfeeding initiation by mothers in the first 48 h after giving birth, a 95 % confidence level, and a 5 % confidence limit. The sample size calculated was 560 infants, which we approximated to 600 infants.

In the city of Tabuk, maternity and child care is provided through a network of 21 primary health care centers (PHCCs). PHCC services attract the majority of the population, who are generally members of the lower and medium socio-economic classes. Of the 21 PHCCs in Tabuk, five were chosen randomly for this research. A selection was made of one PHCC to represent each geographic region of Tabuk using random systematic sampling. More specifically, one PHCC located in each of the north, south, east, west and central areas of Tabuk was randomly selected after stratification by population density, socioeconomic status and geographical location was carried out. The five PHCCs selected for this study each received a letter from Tabuk’s regional health administrator which included a fact sheet summarising the study’s objectives, and inviting health professionals to help by facilitating the completion of the questionnaires.

A convenience sampling method was used to recruit mothers who attended the Well Baby Clinics within the chosen PHCCs with an infant aged ≤ 24 completed months. These eligible mothers were approached in person and invited to an individual interview after they had received a proper orientation. If they agreed to be interviewed, they were supplied with an information sheet and asked to sign a consent form relating to their participation in the study.

### Data collection

In total, 712 mothers were invited to take part in the study; of those, 671 eligible mothers completed the structured questionnaire while attending Well Baby Clinics within the five selected PHCCs, generating a response rate of 94.2 %. They were interviewed by trained, female, Arabic-speaking nurses at each PHCC. Data relating to two main areas were gathered in the study: sociodemographic information and detailed information on breastfeeding practices. The sociodemographic information gathered was as follows: the mother’s age, their education, nationality, employment status, family income, number of children and the mode of delivery of their infant; also, the father’s age, education, nationality and employment status; and the infant’s age, sex, gestational age and birth weight. The detailed information gathered on breastfeeding practices related to the following areas: whether the infants had breastfed within the first 48 h after being born; if so, its duration in months, the frequency of breastfeeding across every 24-h period, the duration of exclusive breastfeeding and the time at which formula was introduced. In cases where the participating mother had more than one child who was younger than two years of age at the time of the research, the information collected from her was focused on her youngest child.

A pilot study involving 25 women was carried out with the purpose of testing the questionnaire which had been designed, so that changes could be made after their feedback had been collected and assessed. We found that the questionnaire took about 15 min to complete, and the pilot study participants were excluded from the final study sample.

### Definitions

In this study, the definition of preterm delivery related to any live infant born at a gestation of < 37 weeks. A low birth weight was taken to refer to any live infant born at a weight of < 2,500 g at birth. The outcome variable (initiation of breastfeeding) was set as having made at least one attempt to breastfeed in the first 48 h after giving birth.

### Data analysis

All the data generated by the questionnaire were assigned appropriate codes, and an analysis was performed on them using the Statistical Package for the Social Sciences (SPSS) program, version 21.0. Univariate associations between various explanatory factors and the initiation of breastfeeding were identified and a multivariate logistic regression analysis was done in order to identify which of the factors had a direct influence on the initiation of breastfeeding, while simultaneously controlling for potential confounders. Odds ratios (OR) and their 95 % confidence intervals (CI) were calculated, *p* ≤ 0.05 was chosen as the level of statistical significance. All the variables which emerged as having significant associations with breastfeeding initiation based on the univariate logistic regression analyses were then entered into the initial multivariate model. Variables which displayed no association with breastfeeding’s initiation were excluded from the final logistic model, provided that they, and their removal from the model, would not have any meaningful effects on the associations between the other variables in the model. This assessment was based on an algorithm originally set out by Hosmer and Lemeshow [21], which has since been used by Tan [17].

## Results

The participant characteristics of the sample (*n* = 671) are described in Table 1. Most of the mothers in the study sample were within the age group of 25–35 years old (60.8 %), and 48.6 % of the mothers had received 12 or more years of schooling. The majority was of Saudi nationality (89.9 %), and the greater proportion was not in employment (78.5 %). With regard to monthly income, the largest group among the mothers (45.3 %) had a household income within the range of 5000–10000 Saudi Riyals per month and 63.5 % of the mothers in this study were multiparous. The majority of births were vaginal deliveries (74.8 %) and 90 % of the infants were born full term, and 11.8 % of the mothers had gestational diabetes.

**Table 1:** Participant characteristics (*n* = 671)

Characteristics of mothers		n	(%)
Age			
	< 25 years	161	24.0
	25–35 years	408	60.8
	> 35 years	102	15.2
Education			
	< 12 years	345	51.4
	≥ 12 years	326	48.6
Nationality			
	Saudi	603	89.9
	Non-Saudi	68	10.1
Employment			
	Employed	144	21.5
	Not-employed	527	78.5
Monthly household income			
	< 5000 SR	199	29.7
	5000–10000 SR	304	45.3
	10,001–15, 000 SR	133	19.8
	> 15,000 SR	35	5.2
Number of children			
	One child	245	36.5
	More than one	426	63.5
Mode of delivery			
	Vaginal	502	74.8
	Caesarean section	169	25.2
Gestation			
	Pre term infant	35	5.2
	Full term infant	636	94.8
Gestational diabetes			
	Yes	79	11.8
	No	587	87.5
	Do not know	5	0.7
Characteristics of fathers			
Age			
	< V25 years	7	1.0
	25–35 years	384	57.2
	> 35 years	280	41.7
Education			
	< 12 years	403	60.1
	≥ 12 years	268	39.9
Nationality			
	Saudi	605	90.2
	Non-Saudi	66	9.8
Employment			
	Employed	610	90.9
	Not-employed	61	9.1
Characteristics of Infants			
Age			
	0–3 months	39	5.8
	4–6 months	129	19.2
	7–9 months	67	10.0
	10–12 months	201	30.0
	13–15 months	38	5.7
	16–18 months	85	12.7
	19–21 month	18	2.7
	22–24 months	94	14.0
Gender			
	Male	357	53.2
	Female	314	46.8
Birth weight			
	Low birth weight	65	9.7
	Normal birth weight	606	90.3

*Yrs* years, *SR* Saudi Riyal, *mos* months

Table 2 contains the results of the univariate and multivariate logistic regression analysis used to identify the variables influencing the initiation of breastfeeding. The proportion of mothers who initiated the breastfeeding within the first 48 h after childbirth was 92.7 %. Among the sociodemographic variables in the univariate logistic regression analysis, the following factors were revealed to have been negatively associated with the initiation of breastfeeding: delivery by caesarean section (Odds Ratio [OR] 0.29, 95 % CI 0.16, 0.52), mothers with infants who were born preterm (OR 0.23, 95 % CI 0.10, 0.54) and infants who were born with a low birth weight (OR 0.33, 95 % CI 0.16, 0.68). The multivariate logistic regression analysis indicated that initiation of breastfeeding within 48 h was less likely in mothers who had given birth by caesarean section (Adjusted Odds Ratio [AdjOR] 0.31, 95 % CI 0.17, 0.57), and who had preterm (AdjOR 0.29, 95 % CI 0.12, 0.70) or low birth weight infants (AdjOR 0.35, 95 % 0.17, 0.75).

**Table 2:** Factors associated with the initiation of breastfeeding within the first 48 h of the infant’s life (*n* = 671)

Variables	Initiation of breastfeeding		Univariate		Multivariate	
	Yes *n* = 622 (92.7 %)	No *n* = 49 (7.3 %)	OR	95 % CI	AdjOR	95 % CI
Mother’s age						
< 25 years	155 (96.3)	6 (3.7)	1.05	0.29, 3.83		
25–35 years	369 (90.4)	38 (9.3)	0.39	0.14, 1.11		
> 35 years	98 (96.1)	4 (3.9)	1	Ref		
Mother’s education						
< 12 years	320 (92.8)	24 (7.0)	1.02	0.57, 1.82		
≥ 12 years	302 (92.6)	24 (7.4)	1	Ref		
Mother’s nationality						
Saudi	557 (92.4)	45 (7.5)	0.56	0.17, 1.85		
Non-Saudi	65 (95.6)	3 (4.4)	1	Ref		
Mother’s dmployment						
Employed	128 (88.9)	15 (10.4)	0.53	0.29, 1.00		
Not-employed	494 (93.7)	33 (6.3)	1	Ref		
Monthly household income						
< 5000 SR	186 (93.5)	13 (6.5)	0.87	0.19, 4.02		
5000–10000 SR	275 (90.5)	28 (9.2)	0.58	0.13, 2.52		
10,001–15, 000 SR	128 (96.2)	5 (3.8)	1.55	0.29, 8.36		
> 15,000 SR	33 (94.3)	2 (5.7)	1	Ref		
Number of children						
One child	226 (92.2)	18 (7.3)	0.90	0.50, 1.64		
More than one	396 (93.0)	30 (7.0)	1	Ref		
Mode of delivery						
Caesarean section	144 (85.2)	25 (14.8)	0.29	0.16, 0.52	0.31	0.17, 0.57
Vaginal	478 (95.2)	23 (4.6)	1	Ref	1	Ref
Gestation						
Preterm infant	27 (77.1)	8 (22.9)	0.23	0.10, 0.54	0.29	0.12, 0.70
Full term infant	595 (93.6)	40 (6.3)	1	Ref	1	Ref
Infant’s gender						
Male	333 (93.3)	24 (6.7)	1.20	0.67, 2.15		
Female	289 (92.0)	24 (7.6)	1	Ref		
Infant birth weight						
Low birth weight	54 (83.1)	11 (16.9)	0.33	0.16, 0.68	0.35	0.17, 0.75
Normal birth weight	568 (93.7)	37 (6.1)	1	Ref	1	Ref

*CI* Confidence Interval, *OR* Odds Ratio, *AdjOR* Adjusted Odds Ratio, *Ref* Reference

## Discussion

Of the 671 mothers surveyed in this study, 92.7 % had initiated breastfeeding within the first 48 h after childbirth, a figure which is consistent with the findings of a recent review of breastfeeding in Saudi Arabia [20]. The authors of that review reported high initiation rates of between 90.1 and 98.9 % in most of the identified studies.

Breastfeeding initiation rates vary across the different countries in the Middle Eastern region. Ninety-five per cent of mothers in the Emirate of Abu Dhabi reported that they initiated breastfeeding after giving birth [22]. This finding is close to that of a research study in Kuwait [23] which reported that the rate of breastfeeding initiation at hospital discharge was 92.5 %, but the figure for Qatar [24] was much lower as only 57 % of mothers reported an early initiation of breastfeeding. A more recent study in Iran reported that 100 % of mothers had started breastfeeding in hospital [25]. Other studies have shown that in Jordan [26], 74 % of new mothers initiated breastfeeding within two hours of giving birth, but only 18.3 % of new mothers in Lebanon started to breastfeed in the first 30 min after giving birth [27].

The present research has not found any significant association between the initiation of breastfeeding in the first 48 h after delivery and the demographic attributes of the mother’s age, education, nationality, employment status, family income, number of children, or infant’s gender. By way of comparison, several studies in the Middle East and in other countries around the world have previously examined the possible associations between the initiation of breastfeeding and some or all of these characteristics, but significant associations have not always been found. A recent review of 15 studies examining breastfeeding in Saudi Arabia summarised that, in terms of the effects of maternal age on breastfeeding, only eight of the studies found a significant association as the breastfeeding initiation rate was higher and breastfeeding was, on average, done for longer by older mothers [20]. Elsewhere, no significant relationship was found between the age of the mother and their breastfeeding practice by a study in Kuwait [23] or by a study in the United Arab Emirates [28]. In another systematic review of 18 research studies from Asia, Africa and South America each of which aimed to identify independent risk factors preventing breastfeeding within the first hour of life, neither the age of the mother nor other factors such as family income or schooling were found to be independent determinants by most of the studies [11].

This study has identified three factors as having statistically significant associations with breastfeeding initiation. The first of these factors, delivery by caesarean section, was inversely associated with breastfeeding initiation. This is consistent with previous studies in Saudi Arabia [13, 29, 30], which identified caesarean section as a risk factor for breastfeeding initiation. For instance, Albokhary and James examined a sample from the western region of Saudi Arabia and found that mothers who had given birth vaginally had a greater likelihood of starting breastfeeding within 24 h, while caesarean section babies were more likely to be formula fed [29]. This negative association of caesarean delivery with breastfeeding initiation has also been reported in a study of Kuwaiti mothers [23] and by a systematic review of worldwide studies which found that 11 out of 14 studies supported the association [11]. Two possibilities have been proposed for this negative association: that the effects of anesthesia delay lactation, and/or post-surgical pain [29, 30].

The present study also identified two other factors which had significant associations with the initiation of breastfeeding. These were gestational age and the infant’s birth weight. More specifically, breastfeeding is less likely to be initiated by mothers with preterm infants and those with infants born at a low birth weight. To the best of the author’s knowledge, only one study previously published in Saudi Arabia has examined gestational age and infant birth weight as possible influencing factors, and found them to be potentially detrimental to breastfeeding initiation [31]. A systematic review [11] was carried out to identify the risk factors likely to lead to breastfeeding not being started within an hour after an infant’s birth. In total, 18 articles met the inclusion criteria, and they were carried out in Asia (9), Africa (5), and South America (4). Only three of them examined the possible association of premature birth with non-breastfeeding, and of those, two studies found that infants born prematurely had significantly less chance of rapidly initiated breastfeeding than full term infants. The remaining study found no association between prematurity and breastfeeding. Further, only five of the 18 studies investigated the possible connection between infants’ birth weights and breastfeeding initiation. Of those five, one identified an association, while the other four did not.

As is the case with all such studies, this study contains some potential limitations. This study’s first, and most important, limitation is the cross-sectional design of the research. This design does not allow causal inferences to be drawn from any of the associations which are found to exist between the identified determinant factors and breastfeeding initiation. A cross-sectional design also has the inherent limitation of recall bias, especially with regard to mothers who have older infants, as their recollections may be less accurate. The study has a second limitation in that it only used mothers who were attending governmental PHCCs, who were interested in breastfeeding and, therefore, in participating. It also excluded mothers who were receiving similar care at other health facilities, including those in the private sector. Overall, the nature of the sample might be said to restrict the generalizability of the study.

## Conclusions

The key finding of this study is that the initiation of breastfeeding within 48 h was less likely to be reported by mothers who had given birth by caesarean section, and had preterm or low birth weight infants. These variables each present important barriers to breastfeeding initiation, which hospitals could assist in overcoming. Suitable hospital policies and staff training must therefore be implemented in order to support mothers in initiating breastfeeding soon after giving birth, and to discourage them from giving their babies infant formula unnecessarily in hospital. A particular focus should be placed on providing additional support to mothers identified as being at a higher risk of failing to initiate breastfeeding, so that higher rates of exclusive breastfeeding can be encouraged and achieved in Saudi Arabia.
